# Degree of food processing and breast cancer risk: a prospective study in 9 European countries

**DOI:** 10.1186/s43014-024-00264-2

**Published:** 2024-10-09

**Authors:** Manon Cairat, Sahar Yammine, Thibault Fiolet, Agnès Fournier, Marie-Christine Boutron-Ruault, Nasser Laouali, Francesca Romana Mancini, Gianluca Severi, Fernanda Morales Berstein, Fernanda Rauber, Renata Bertazzi Levy, Guri Skeie, Kristin Benjaminsen Borch, Anne Tjønneland, Lene Mellemkjær, Yan Borné, Ann H. Rosendahl, Giovanna Masala, Maria Teresa Giraudo, Maria Santucci de Magistris, Verena Katzke, Rashmita Bajracharya, Carmen Santiuste, Pilar Amiano, Stina Bodén, Carlota Castro-Espin, Maria-Jose Sánchez, Mathilde Touvier, Mélanie Deschasaux-Tanguy, Bernard Srour, Matthias B. Schulze, Marcela Guevara, Nathalie Kliemann, Jessica Blanco Lopez, Aline Al Nahas, Kiara Chang, Eszter P. Vamos, Christopher Millett, Elio Riboli, Alicia K. Heath, Carine Biessy, Vivian Viallon, Corinne Casagrande, Genevieve Nicolas, Marc J. Gunter, Inge Huybrechts

**Affiliations:** 1 Nutrition and Metabolism Branch, International Agency for Research on Cancer, Lyon, France; 2 Université Paris-Saclay, UVSQ, Inserm, Gustave Roussy, CESP, 94805 Villejuif, France; 3 Université Sorbonne Paris Nord and Université Paris Cité, INSERM, INRAE, CNAM, Center of Research in Epidemiology and StatisticS (CRESS), Nutritional Epidemiology Research Team (EREN), F-93017 Bobigny, France; 4 Population Health Sciences, Bristol Medical School, University of Bristol, Bristol, United Kingdom; 5 MRC Integrative Epidemiology Unit, University of Bristol, Bristol, United Kingdom; 6 Department of Preventive Medicine, School of Medicine, University of São Paulo, São Paulo, Brazil; 7 Department of Community Medicine, Faculty of Health Sciences, UiT – the Arctic University of Norway, Tromsø, Norway; 8 Danish Cancer Society Research Center, Copenhagen, Denmark; 9 Department of Public Health, University of Copenhagen, Copenhagen, Denmark; 10 Nutrition Epidemiology, Department of Clinical Sciences Malmö, Faculty of Medicine, Lund University, Lund, Sweden; 11 Department of Clinical Sciences Lund, Oncology, Lund University and Skåne University Hospital, Lund, Sweden; 12 Institute for Cancer Research, Prevention and Clinical Network (ISPRO), Florence, Italy; 13 Department of Clinical and Biological Sciences and Centre for Biostatistics, Epidemiology, and Public Health (C-BEPH), Turin, Italy; 14 Dipartimento di medicina clinica e chirurgia Federico ii university, Naples, Italy; 15 Department of Cancer Epidemiology, German Cancer research Center (DKFZ), Heidelberg, Germany; 16 Department of Epidemiology, Murcia Regional Health Council, IMIB-Arrixaca, Murcia, Spain; 17 Spanish Consortium for Research on Epidemiology and Public Health (CIBERESP), Instituto de Salud Carlos III, Madrid, Spain; 18 Epidemiology of Chronic and Communicable Diseases Group, Biodonostia Health Research Institute, San Sebastián, Spain; 19 Sub Directorate for Public Health and Addictions of Gipuzkoa, Ministry of Health of the Basque Government, San Sebastian, Spain; 20 Department of Clinical Sciences, Pediatrics, Umeå University, Umeå, Sweden; 21 Unit of Nutrition and Cancer, Cancer Epidemiology Research Program, Catalan Institute of Oncology (ICO), Av. Granvia de L’Hospitalet 199-203, L’Hospitalet de Llobregat, Barcelona, Spain; 22 Unit of Nutrition and Cancer, Epidemiology, Public Health, Cancer Prevention and Palliative Program, Bellvitge Biomedical Research Institute (IDIBELL), L’Hospitalet de Llobregat, Av. Granvia de L’Hospitalet, 199-203 Barcelona, Spain; 23 Escuela Andaluza de Salud Pública (EASP), 18011 Granada, Spain; 24 Instituto de Investigación Biosanitaria ibs.GRANADA, 18012 Granada, Spain; 25 Department of Preventive Medicine and Public Health, University of Granada, 18071 Granada, Spain; 26 Nutrition And Cancer Research Network (NACRe Network), Paris, France; 27 Department of Molecular Epidemiology, German Institute of Human Nutrition Potsdam-Rehbruecke, Nuthetal, Germany; 28 Institute of Nutritional Science, University of Potsdam, Nuthetal, Germany; 29 Instituto de Salud Pública y Laboral de Navarra, 31003 Pamplona, Spain; 30 Navarra Institute for Health Research (IdiSNA), 31008 Pamplona, Spain; 31 Cancer Hospital and Research Centre of Santa Catarina (CEPON), Florianópolis, Brazil; 32 Public Health Policy Evaluation Unit, School of Public Health, Imperial College London, London, United Kingdom; 33 Department of Epidemiology and Biostatistics, School of Public Health, Imperial College London, London, United Kingdom; 34 Inserm U1018, “Exposome, Heredity, Cancer, and Health” Team, Hôpital Paul Brousse, Bâtiment 15/16, 16 avenue Paul Vaillant Couturier, 94807 VILLEJUIF CEDEX, France

**Keywords:** Epidemiology, Prospective study, Breast cancer, NOVA classification, Food processing

## Abstract

Recent epidemiological studies have suggested a positive association between ultra-processed food consumption and breast cancer risk, although some studies also reported no association. Furthermore, the evidence regarding the associations between intake of food with lower degrees of processing and breast cancer risk is limited.

Thus, we investigated the associations between dietary intake by degree of food processing and breast cancer risk, overall and by breast cancer subtypes in the European Prospective Investigation into Cancer and Nutrition (EPIC) study.

Dietary intake of EPIC participants was assessed via questionnaires at baseline. More than 11,000 food ingredients were classified into four groups of food processing levels using the NOVA classification system: unprocessed/minimally processed (NOVA 1), culinary ingredients (NOVA 2), processed (NOVA 3) and ultra-processed (NOVA 4). Cox proportional hazards models were used to estimate hazard ratios (HRs) and 95% confidence intervals (CIs) of breast cancer per standard deviation increase in daily consumption (grams) of foods from each NOVA group.

The current analysis included 14,933 breast cancer cases, diagnosed among the 318,686 EPIC female participants, (median follow-up of 14.9 years). No associations were found between breast cancer risk and the level of dietary intake from NOVA 1 [HR _per 1 SD_=0.99 (95% CI 0.97 – 1.01)], NOVA 2 [HR _per 1 SD_ =1.01 (95% CI 0.98 – 1.03)] and NOVA 4 [HR _per 1 SD_ =1.01 (95% CI 0.99 – 1.03)] foods. However, a positive association was found between NOVA 3 and breast cancer risk [HR _per 1 SD_ =1.05 (95% CI 1.03 – 1.07)] which became non-significant after adjustment for alcohol intake [HR _per 1 SD_ =1.01 (95% CI 0.98 – 1.05)] or when beer and wine were excluded from this group [HR _per 1 SD_ =0.99 (95% CI 0.97 – 1.01)]. The associations did not differ by breast cancer subtype, menopausal status or body mass index. Findings from this large-scale prospective study suggest that the positive association between processed food intake and breast cancer risk was likely driven by alcoholic beverage consumption.

**Graphical Abstract:**

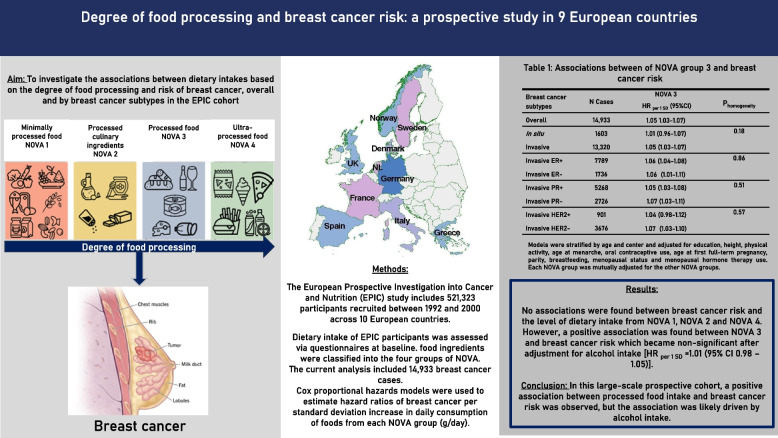

**Supplementary Information:**

The online version contains supplementary material available at 10.1186/s43014-024-00264-2.

## Introduction

Diets have transitioned from fresh, unprocessed, and minimally processed foods toward a rise in the consumption of ultra-processed foods. These now contribute roughly 25 to 60% of the total daily energy intake of individuals across countries (Adams & White, 2015; Juul et al., 2022; Latasa et al., 2018; Levy et al., 2022; Madruga et al., 2022; Marrón-Ponce et al., 2019; Moubarac et al., 2014a; Wang et al., 2021). In the past decades, several food processing frameworks have been developed, such as the NOVA classification, the Food Standards Australia New Zealand, the International Food and Information Council, and the International Food Policy and Research Institute (Bleiweiss-Sande et al., 2019; Crino et al., 2017; Moubarac et al., 2014b). Although most of these similarly classify basic foods as processed or unprocessed, the NOVA system is the most widely applied to scientific studies and may be more useful for monitoring changes in the food supply and evaluating associations with health outcomes (Crino et al., 2017; Monteiro et al., 2019). The NOVA classification categorizes foods into four groups based on the extent and purpose of food processing they undergo: unprocessed/minimally processed foods (NOVA 1), processed culinary ingredients (NOVA 2), processed foods (NOVA 3) and ultra-processed foods (NOVA 4) (Monteiro et al., 2019). Foods classified as NOVA 4, which are foods that undergo multiple physical, biological, and/or chemical processes, have been suggested to have detrimental health effects due to their poorer nutritional qualities on average (e.g. they are often energy dense and/or rich in saturated and trans-fatty acids) or the presence of a wide range of additives and contaminants formed during processing (Lane et al., 2021; Pagliai et al., 2021; Srour et al., 2022). Epidemiological studies investigating the association between the consumption of foods classified by the NOVA scale and the risk of breast cancer are sparse. Breast cancer risk was inversely associated with NOVA 1 (Fiolet et al., 2018; Jacobs et al., 2022), positively associated with NOVA 3 (Kliemann et al., 2023), and either not associated (Jacobs et al., 2022; Romaguera et al., 2021) or positively (Chang et al., 2023; Fiolet et al., 2018; Romieu et al., 2022) associated with NOVA 4. A recent analysis in the European Prospective Investigation into Cancer and Nutrition (EPIC), performed by our group, suggested that higher consumption of NOVA 1 was associated with lower breast cancer risk while higher consumption of NOVA 3 was associated with higher breast cancer risk (Kliemann et al., 2023). In that study, we did not stratify our analyses by breast cancer subtype although such analyses could shed light on the potential mechanisms underlying the associations observed between different degrees of food processing and breast cancer, since the etiologies behind breast cancer subtypes are different. Furthermore, few previous studies were able to stratify their analyses by breast cancer subtypes or by alcohol intake and body mass index (BMI). Therefore, the aim of this study was to investigate the associations between diet according to the degree of food processing and breast cancer risk, overall and by breast cancer subtype, menopausal status, alcohol intake and BMI, within the EPIC cohort.

## METHODS

### EPIC Cohort

Between 1992 and 2000, a total of 521,323 EPIC participants were recruited from 23 centers across 10 European countries (Riboli et al., 2002). At recruitment, socio-demographic, dietary, lifestyle, anthropometric and medical data were collected for all participants by administration of validated country-specific questionnaires. Ethical approval for the study was obtained from the relevant ethical review boards of the participating centers of EPIC as well as from the ethics committee of the International Agency for Research on Cancer (IARC).

### Study population and follow-up

The selection of the study population is shown in Supplementary Figure S1. We used data from all participating countries apart from Greece due to a lack of data access. Participants were further excluded if they (i) had any cancer diagnosis before recruitment, (ii) had no follow-up, (iii) had no lifestyle or dietary information, (iv) had an energy intake-to-requirement ratio within the extreme ranking (top and bottom 1%, which are implausible dietary exposure values), or (v) were men. Women were followed from study inclusion until the date of their latest known contact, cancer diagnosis, death, emigration, or the end of the follow-up period (between 2008 and 2014 depending on the center), whichever occurred first. The analytical sample included 318,686 women who were free of cancer at recruitment.

### Identification of incident breast cancer cases

In Italy, Spain, the United Kingdom, the Netherlands, Denmark, Norway, and Sweden, population-based cancer registries were used to identify breast cancer cases. In France and Germany, a combination of methods was used, including health insurance records, contacts with cancer and pathology registries, and active follow-up of participants and their next of kin. Almost all centers (except Malmö, Granada, and Murcia) had information on tumor characteristics, including invasiveness status (*in situ*/invasive/unknown), estrogen receptor (ER) status (ER-positive/ER-negative/unknown), progesterone receptor (PR) status (PR-positive/PR-negative/unknown), and human epidermal growth factor receptor 2 (HER2) status (HER2-positive/HER2-negative/unknown). The diagnosis of breast cancer cases was based on the 2nd or the 3rd revision (depending on the year of diagnosis) of the International Classification of Diseases for Oncology (ICD-O-2 or ICD-O-3) (International Statistical Classification of Diseases and Related Health Problems 10th Revision, n.d.). In the present work, the first diagnosis of breast cancer was identified as primary incident breast tumors. Vital status was collected from regional or national mortality registries.

### Dietary data and NOVA classification

Country-specific dietary questionnaires were used in EPIC and validated at the center level (Huybrechts et al., 2022). Semi-quantitative food frequency questionnaires, extensive quantitative dietary questionnaires, and combined methods (i.e. a 7-day record on hot meals was combined with quantitative food-frequency questionnaires in Malmö, Sweden) were used to collect dietary data at baseline. These were center specific to account for local dietary habits and were either self-administered or administered in-person by trained interviewers. These data were then harmonized to obtain a standardized food list with comparable detail across countries. The dietary questionnaires and their mode of administration were described in detail in previous publications (Riboli et al., 2002, Huybrechts I et al., 2022). Then, more than 11,000 foods/ingredients/beverages were categorized into one of the four NOVA groups based on their degrees of food processing. The different NOVA groups are defined in the Additional File (Text S1). The classification of EPIC foods into NOVA groups has been described in depth elsewhere (Huybrechts et al., 2022). To account for potential changes in industrialization over time, lower, middle, and upper bound scenarios were created. The "middle bound" scenario, deemed most likely in the past 25 years, was used for the primary analysis. In the “lower bound” scenario, foods with potential for less processing were assigned to a lower processed NOVA group, while in the “upper bound” scenario, foods with potential for more processing were assigned to a higher processed.

For each study participant, we calculated the dietary intake from each NOVA group as expressed by (1) the total absolute intake in grams/day (g/day) and (2) the total absolute intake in kcal/day (kcal/d). We also calculated the relative contribution of each NOVA food group to the total daily dietary intake in grams (%g/day) and kcal (%kcal/day). The g/d unit was considered the primary exposure because it better captures industrial foods with zero calorie content (e.g., artificially sweetened drinks) and food processing factors (e.g., neoformed contaminants or food additives).

#### Covariates at recruitment

Information on lifestyle, reproductive/hormonal factors, and medical history was gathered using baseline questionnaires. All EPIC centers collected information on educational level, age at menarche, age at first full term pregnancy and parity, breastfeeding, and use of oral contraceptives and menopausal hormone therapy (MHT). Menopausal status was determined by combining different baseline information. Women who reported to have menstrual cycles, had at least nine menstrual periods over the previous 12 months, or were younger than 42 years were considered as premenopausal women. Women who reported fewer than four menses in the past year, a bilateral ovariectomy, or were older than 55 years were considered as postmenopausal women. Otherwise, women were considered perimenopausal.

Body weight and height were either measured by a health care professional or self-reported in each center. Weight and height were used to calculate BMI defined as weight in kilograms divided by height in meters squared (kg/m^2^). Physical activity levels were estimated using a questionnaire focused on past-year physical activity in occupational, leisure, and household domains. The Cambridge physical activity index was then created by combining occupational physical activity with time spent in physical exercise (such as cycling, swimming, and jogging) (Wareham et al., 2003). Alcohol intake in grams per day was based on the number of standard glasses of wine, beer, cider, sweet liquor, distilled spirits, or fortified wines consumed daily or weekly during the 12 months before recruitment. The Mediterranean diet score was calculated using a methodology previously described (Couto et al., 2011).

### Statistical analysis

In the main analyses, the middle-bound scenario for the NOVA classification and the absolute g/d of the four NOVA food groups were used.

Baseline characteristics were examined by quartiles of each NOVA food group intake. Multivariable Cox proportional hazards regression models were performed to estimate hazard ratios (HRs) and their corresponding 95% confidence intervals (CIs) for the associations between the intake of each NOVA food group [1 standard deviation (SD) increment] and breast cancer incidence, overall and by breast cancer subtypes. Age served as the primary time scale. All four NOVA groups were simultaneously included in the Cox model.

All models were stratified by age at recruitment in 1-year categories and study center and adjusted for potential confounding factors including educational level (none, primary school, technical/professional school, higher education), physical activity (inactive, moderately inactive, moderately active, active), height in cm (continuous), age at menarche in years (≤ 13, > 13), oral contraceptive use (never, ever, unknown), pregnancies (nulliparous,1 or 2 children, >3 children), age at first full-term pregnancies (continuous), breastfeeding (never, ever, unknown), menopausal status (pre, peri, post-menopause), and menopausal hormone therapy (MHT) use (never, ever, unknown). We investigated whether adding different dietary-related factors/components (BMI, total energy intake, total fat, sodium intake, carbohydrate intake, Mediterranean diet, and alcohol intake) to the model changed the HR associated with NOVA groups. Only alcohol consumption modified the HRs and was therefore included in an additional model (Supplementary Table S1). Ever use of oral contraceptives and MHT and breastfeeding had >5% missing values, which were accommodated by using a “missing” category in the models. All other covariates had <5% missing values, which were replaced with the mode for categorical variables, or the median for continuous variable values observed among the subjects with complete data.

Heterogeneities according to the invasiveness status or hormonal receptor status were evaluated with competing risk analyses. In these analyses, cases with missing information on the studied subtype were excluded from the corresponding analysis and those who developed the competing breast cancer subtypes were censored at the time of occurrence (Lunn & McNeil, 1995). Heterogeneities were calculated as the deviations of logistic beta-coefficients observed in each of the subgroup relative to the overall beta-coefficient.

As subgroup and sensitivity analyses, we repeated the analyses (1) by using lower and upper bound scenarios for the NOVA classification, (2) by using consumption of NOVA groups measured as %g/d, kcal/d and %kcal/d instead of g/d, and (3) by removing alcoholic drinks (present in NOVA groups 3 and 4). When we analyzed the intake of NOVA food groups measured as the proportion of overall daily food intake (%g/d) or daily kcal intake (%kcal/d), Cox regression analyses were performed separately for each NOVA group. Finally, we explored whether associations between NOVA groups and breast cancer risk varied by alcohol intake, BMI categories, menopausal status and country. Effect modification was evaluated by using likelihood ratio tests to compare models with and without cross-product interaction terms. Statistical analyses were conducted using SAS software (version 9.4, Copyright © 2017, SAS Institute Inc.).

## Results

During a median follow-up time of 14.9 years (13.5-16.4), 14,933 breast cancer cases were diagnosed (1,603 *in situ*, 13,320 invasive, and 10 of unknown invasiveness status) among the 318,686 participants. Among the invasive breast cancer cases, 9,525 had information on ER status (7,789 ER-positive and 1,736 ER-negative), 7,994 on PR status (5,268 PR-positive and 2,726 PR-negative), and 4,577 on HER2 status (901 HER2-positive and 3,676 HER2-negative). There were 573 ER+PR±HER2+, 3023 ER+PR±HER2-, 264 ER-PR-HER2-, and 419 ER-PR-HER2+ breast cancer cases.

Using the middle-bound scenario expressed as g/d, consumption of food classified as NOVA 1 contributed 74% of the total diet (Table 1). The main foods contributing to this group were coffee/tea (31%), water (19%), fruits (12%) and milk/plain yogurt (12%) (Table 2). The contribution of NOVA 2 to the total diet was 1%, with plant oils being the highest contributor to the group (37%) followed by table sugar (29%), and animal fats (28%). The contribution of processed foods (NOVA 3) to the total diet was 11%, with an important contribution of beer/wine (35%) and processed bread (26%). Overall, ultra-processed foods (NOVA 4) contributed 13% to the total diet with dairy desserts and drinks among the top group contributors (14%), followed by soft drinks (13%), ultra-processed breads (12%) and sweetened beverages (11%). The relative intake of food classified as NOVA 1 was highest in France (80%) and Denmark (79%). The relative intake of food classified as of NOVA 2 was highest in Italy (3%) and Spain (2%). NOVA 3 foods were mostly highly consumed in Italy (23%), Spain (14%) and Germany (14%), while NOVA 4 foods were highly consumed in Norway (23%) and the United Kingdom (19%).

**Table 1:** NOVA group intake and relative and absolute contributions to total diet overall and by country.

**NOVA group** Country		**g/d**		**%g/d**	
	**N**	**Mean**	**SD**	**Mean**	**SD**
**NOVA 1**					
All	318,686	1979.9	853.5	74.2	10.7
France	67,403	2492.1	796.2	79.6	8.0
Italy	30,513	1113.9	361.9	63.7	10.1
Spain	24,850	1344.5	379.8	75.4	10.3
The United Kingdom	52,566	2068.3	621.4	74.3	10.3
The Netherlands	26,912	2216.5	619.3	76.3	8.3
Germany	27,379	1921.3	787.9	69.3	11.0
Sweden	26,368	1984.4	762.8	77.6	8.1
Denmark	28,720	2849.4	802.3	78.7	8.9
Norway	33,975	1190.6	381.1	68.1	9.7
**NOVA 2**					
All	318,686	27.0	21.6	1.2	1.0
France	67,403	42.8	19.4	1.4	0.7
Italy	30,513	47.4	19.7	2.8	1.0
Spain	24,850	37.5	18.5	2.1	1.0
The United Kingdom	52,566	13.9	14.8	0.5	0.6
The Netherlands	26,912	18.5	17.9	0.7	0.7
Germany	27,379	25.7	20.8	1.0	0.8
Sweden	26,368	20.9	18.1	0.9	0.7
Denmark	28,720	15.1	12.3	0.4	0.4
Norway	33,975	12.5	8.7	0.7	0.5
**NOVA 3**					
All	318,686	278.0	190.5	11.3	7.7
France	67,403	356.5	196.7	11.9	6.4
Italy	30,513	399.2	189.8	23.0	9.2
Spain	24,850	254.9	169.0	14.2	8.2
The United Kingdom	52,566	182.3	146.9	6.7	4.8
The Netherlands	26,912	248.4	137.1	8.8	4.6
Germany	27,379	366.5	202.5	13.9	6.8
Sweden	26,368	238.4	139.3	9.5	4.7
Denmark	28,720	302.2	220.0	8.5	5.9
Norway	33,975	140.9	74.5	8.3	4.2
**NOVA 4**					
All	318,686	333.2	240.8	13.3	8.9
France	67,403	215.5	135.6	7.2	4.5
Italy	30,513	184.6	138.9	10.5	6.6
Spain	24,850	146.4	125.5	8.2	6.5
The United Kingdom	52,566	508.5	296.0	18.5	9.3
The Netherlands	26,912	403.6	200.9	14.2	6.7
Germany	27,379	417.9	254.3	15.9	8.8
Sweden	26,368	294.1	172.8	12.0	6.2
Denmark	28,720	434.4	280.0	12.3	7.3
Norway	33,975	386.9	163.2	22.8	8.8

*NOVA 1* Unprocessed/minimally processed foods, *NOVA 2* Processed culinary ingredients, *NOVA 3* Processed foods, *NOVA 4* Ultra -processed foods, *SD* Standard Deviation

**Table 2:** Absolute intakes and relative contributions of food to total diet and to each NOVA group

**NOVA groups**	**Food groups**	**g/d**		**% in total diet**		**% within the food group**
		**Mean**	**SD**	**Mean**	**SD**	**Mean**
**1**	**Water**	382.23	531.38	11.81	14.65	19.3
	**Fruit**	232.79	176.68	9.68	7.68	11.8
	**Milk and plain yoghurt**	234.29	205.38	9.22	7.63	11.8
	**Cereal, grains and flour made from these foods**	40.89	50.14	1.67	1.95	2.1
	**Potatoes**	75.8	58.63	3.15	2.65	3.8
	**Fresh pasta**	34.52	40.19	1.57	2.15	1.7
	**Beans, lentils and chickpeas**	18.47	24.06	0.79	1.14	0.9
	**Vegetables**	186.24	117.89	7.54	4.74	9.4
	**Nuts and Seeds**	2.28	5.22	0.09	0.21	0.1
	**Eggs**	17.55	16.37	0.72	0.66	0.9
	**Poultry**	17.3	17.63	0.75	0.86	0.9
	**Red meat**	41.65	34.35	1.7	1.44	2.1
	**Fish**	23.56	27.11	1.07	1.47	1.2
	**Seafood**	3.01	5.56	0.14	0.26	0.2
	**Fungi**	5.93	8.81	0.22	0.32	0.3
	**Coffee/tea**	606.68	462.33	21.97	13.6	30.6
	**Fruit juice fresh and smoothies**	10.58	25.65	0.4	1.02	0.5
	**Fruit juice UHT or pasteurised**	42.78	70.3	1.66	2.75	2.2
	**Homemade broth**	3.31	7.93	0.11	0.28	0.2
**2**	**Table sugar**	7.8	11.62	0.33	0.51	28.9
	**Plant oil**	9.97	11.95	0.46	0.62	36.9
	**Animal fats**	7.52	9.76	0.3	0.39	27.9
	**Other processed culinary ingredients**	1.3	2.71	0.04	0.09	4.8
	**Table salt**	0.4	0.65	0.01	0.02	1.5
**3**	**Cheese**	37.35	33.57	1.54	1.45	13.4
	**Salted, smoked or canned meat, without additives**	5.83	12.64	0.28	0.7	2.1
	**Salted, smoked or canned fish**	6.66	9.69	0.28	0.42	2.4
	**Processed bread**	72.83	80.59	3.14	3.76	26.2
	**Vegetables and other plant foods preserved**	16.49	26.1	0.69	1.2	5.9
	**Legumes preserved**	4.41	11.32	0.19	0.56	1.6
	**Fruit preserved**	17.8	25.28	0.68	0.94	6.4
	**Nuts salted and nut spreads**	1.57	4.28	0.06	0.18	0.6
	**Beer and Wine**	96.93	150.13	3.68	5.31	34.9
	**Condensed milk, yogurt plain sweetened**	6.15	17.53	0.24	0.66	2.2
	**Bread crumbs**	0.21	0.59	0.01	0.02	0.1
	**Meringue, non ultra-processed bakeries**	8.74	17.42	0.37	0.7	3.1
	**Cheese**	3.06	6.1	0.14	0.3	1.1
**4**	**Ultra-processed breads**	40.57	62.77	1.72	2.95	12.1
	**Pastries, buns, and cakes**	21.09	26.95	0.87	1.2	6.3
	**Biscuits**	14.48	18.15	0.63	0.89	4.3
	**Breakfast cereals**	4.62	9.76	0.17	0.37	1.4
	**Ice cream, ice pops and frozen yogurts**	7.11	11.53	0.31	0.56	2.1
	**Industrial desserts**	1.26	8.96	0.05	0.33	0.4
	**Packaged salty snacks**	1.75	5.14	0.07	0.21	0.5
	**Potato products**	6.95	15.08	0.26	0.58	2.1
	**Pizza and focaccia**	6.55	10.41	0.29	0.52	1.9
	**Filled Pasta (industrial pasta)**	3.09	7.96	0.14	0.4	0.9
	**Instant and canned soups**	9	19.67	0.35	0.78	2.7
	**Dairy substitute products**	3.2	32.73	0.13	1.31	1
	**Processed cheese**	3.05	6.68	0.14	0.31	0.9
	**Sauces, dressing and gravies**	9.39	12.12	0.34	0.43	2.8
	**Vegetable spread and products**	0.19	1.18	0.01	0.05	0.1
	**Soft drinks**	43.32	109.71	1.8	4.35	12.9
	**Dairy desserts and drinks**	46.27	69.3	1.77	2.5	13.8
	**Sweetened beverages**	37.88	113.82	1.31	3.53	11.3
	**Beverages (dry weight) (ex. Coffee powder, chocolate powder and milk powder)**	0.86	3.71	0.03	0.14	0.3
	**Alcoholic distilled drinks**	7.5	20.23	0.27	0.71	2.2
	**Artificial sweeteners**	0.48	2.55	0.02	0.08	0.1
	**Sweet snacks**	11.32	18.44	0.43	0.7	3.4
	**Processed meat**	31.76	29.04	1.38	1.47	9.4
	**Meat alternatives**	0.85	3.93	0.03	0.15	0.3
	**Nutrition powders and drinks**	0.01	0.47	0	0.02	0
	**Margarine**	10.6	13.64	0.43	0.59	3.2
	**Ready meals**	5.12	10.77	0.17	0.36	1.5
	**Alcohol-free versions of alcoholic beverages**	1.62	19.3	0.06	0.7	0.5
	**Ultra-processed vegetables and legumes**	3.31	12.18	0.12	0.45	1.0
	**Rice-based dishes**	0	0.13	0	0.01	0

*NOVA 1* Unprocessed/minimally processed foods, *NOVA 2* Processed culinary ingredients, *NOVA 3* Processed foods, *NOVA 4* Ultra-processed foods

The main baseline characteristics of participants by quartiles of intake of NOVA 1, NOVA 2, NOVA 3, and NOVA 4 are presented in supplementary Tables S2, S3, S4 and S5, respectively.

Table 3 shows the associations between the middle-bound scenario of each NOVA group intake (in g/d) and breast cancer, overall and by breast cancer subtypes. Overall, intake of NOVA 1 [HR_per 1 SD_=0.99 (95% CI 0.97 – 1.01)], NOVA 2 [HR_per 1 SD_=1.01 (0.98 – 1.03)], and NOVA 4 [HR_per 1 SD_=1.01 (0.99 – 1.03)] were not associated with breast cancer risk. However**,** intake of processed foods (NOVA 3) was associated with a higher risk of breast cancer [HR_per 1 SD_=1.05 (1.03 – 1.07)]. Estimates did not differ by invasiveness or hormone receptor status (Table 3, P_homogeneity_≥ 0.11). When the model was further adjusted for alcohol intake (Table 4), the positive association between NOVA 3 and breast cancer risk was attenuated and no longer statistically significant [HR_per 1 SD_=1.01 (0.98 – 1.03)]. Furthermore, when alcoholic drinks were excluded from NOVA 3 the association with breast cancer risk was also null [HR_per 1 SD_=0.99 (0.97 – 1.01), Table 5]. Associations were similar when models were stratified by alcohol intake, BMI at recruitment (P_interaction_ ≥ 0.17, Table 6) or menopausal status (Table S6).

**Table 3:** Associations between of NOVA groups (in g/d) and breast cancer risk, overall and by breast cancer subtypes

**Breast cancer subtypes**	**N Cases**	**NOVA 1**		**NOVA 2**		**NOVA 3**		**NOVA 4**	
		HR _per 1 SD_ (95%CI)^a^	P_homogeneity_	HR _per 1 SD_ (95%CI)^a^	P_homogeneity_	HR _per 1 SD_ (95%CI)^a^	P_homogeneity_	HR _per 1 SD_ (95%CI)^a^	P_homogeneity_
Overall	14,933	0.99 (0.97-1.01)		1.01 (0.98-1.03)		1.05 (1.03-1.07)		1.01 (0.99-1.03)	
*In situ*	1603	0.97 (0.91-1.04)	0.63	1.00 (0.94-1.07)	0.87	1.01 (0.96-1.07)	0.18	1.01 (0.99-1.03)	0.87
Invasive	13,320	0.99 (0.97-1.01)		1.01 (0.98-1.03)		1.05 (1.03-1.07)		1.01 (0.95-1.07)	
Invasive ER+	7789	0.99 (0.96-1.02)	0.54	1.00 (0.97-1.03)	0.29	1.06 (1.04-1.08)	0.86	1.00 (0.98-1.03)	0.11
Invasive ER-	1736	1.01 (0.95-1.07)		1.04 (0.98-1.10)		1.06 (1.01-1.11)		1.06 (1.00-1.11)	
Invasive PR+	5268	0.99 (0.96-1.03)	0.56	1.01(0.98-1.04)	0.34	1.05 (1.03-1.08)	0.51	0.99 (0.95-1.02)	0.15
Invasive PR-	2726	0.98 (0.93-1.03)		0.98 (0.93-1.03)		1.07 (1.03-1.11)		1.03 (0.98-1.09)	
Invasive HER2+	901	0.99 (0.91-1.08)	0.97	1.00 (0.92-1.09)	0.70	1.04 (0.98-1.12)	0.57	1.04 (0.97-1.13)	0.39
Invasive HER2-	3676	0.99 (0.95-1.03)		1.02 (0.98-1.06)		1.07 (1.03-1.10)		1.01 (0.97-1.03)	

*CI* Confidence Interval, *ER* Estrogen Receptor, *HER2* Human epidermal growth factor receptor 2, *HR* Hazard ratio, *NOVA 1* unprocessed/minimally processed foods, *NOVA 2* Processed culinary ingredients, *NOVA 3* Processed foods, *NOVA 4* Ultra-processed foods, *PR* Progesterone receptor, *SD* Standard deviation

^a^Models were stratified by age and center and adjusted for education, height, physical activity, age at menarche, oral contraceptive use, age at first full-term pregnancy, parity, breastfeeding, menopausal status and menopausal hormone therapy use. Each NOVA group was mutually adjusted for the other NOVA groups

**Table 4:** Associations between NOVA groups (in g/d) and breast cancer risk with further adjustment for alcohol consumption

**Breast cancer subtypes**	**N Cases**	**NOVA 1**		**NOVA 2**		**NOVA 3**		**NOVA 4**	
		HR per 1 SD (95%CI)^a^	P_homogeneity_	HR per 1 SD (95%CI)^a^	P_homogeneity_	HR per 1 SD (95%CI)^a^	P_homogeneity_	HR per 1 SD (95%CI)^a^	P_homogeneity_
Overall	14,933	0.99 (0.97-1.01)		1.01 (0.99-1.03)		1.01 (0.98-1.03)		1.00 (0.98-1.03)	
*In situ*	1603	0.98 (0.91-1.04)	0.61	1.00 (0.94-1.07)	0.84	0.98 (0.90-1.07)	0.51	1.01 (0.95-1.08)	0.89
Invasive	13,320	0.99 (0.97-1.02)		1.01 (0.99-1.03)		1.01 (0.98-1.04)		1.01 (0.99-1.03)	
Invasive ER+	7789	0.99 (0.96-1.02)	0.58	1.00 (0.97-1.04)	0.33	1.00 (0.97-1.04)	0.50	1.00 (0.98-1.03)	0.11
Invasive ER-	1736	1.01 (0.95-1.08)		1.04 (0.98-1.11)		1.03 (0.96-1.12)		1.06 (1.00-1.11)	
Invasive PR+	5268	1.00 (0.96-1.04)	0.51	1.01 (0.98-1.05)	0.30	1.00 (0.95-1.06)	0.30	0.99 (0.95-1.03)	0.15
Invasive PR-	2726	0.98 (0.93 -1.03)		0.98 (0.94-1.03)		1.04 (0.98-1.11)		1.03 (0.98-1.09)	
Invasive HER2+	901	0.99 (0.91-1.08)	0.91	1.00 (0.92-1.08)	0.56	1.07 (0.96-1.19)	0.29	1.04 (0.97-1.12)	0.44
Invasive HER2-	3676	1.00 (0.95-1.04)		1.02 (0.98-1.07)		1.00 (0.95-1.05)		1.01 (0.97-1.05)	

*CI* Confidence Interval, *ER* Estrogen Receptor, *HER2* Human epidermal growth factor receptor 2, *HR* Hazard ratio, *NOVA 1 *Unprocessed/minimally processed foods, *NOVA 2* Processed culinary ingredients, *NOVA 3* Processed foods, *NOVA 4* Ultra-processed foods; PR, progesterone receptor, *SD* Standard deviation

^a^Models were stratified by age and center and adjusted for education, height, physical activity, age at menarche, oral contraceptive use, age at first full-term pregnancy, parity, breastfeeding, menopausal status, menopausal hormone therapy use and alcohol consumption**.** Each NOVA group was mutually adjusted for the other NOVA groups

**Table 5:** Associations between NOVA 3 intake after excluding alcoholic drinks (in g/d) and breast cancer risk

**Breast cancer subtypes**	**HR **_**per 1 SD**_** (95%CI)**^a^	***P***_***homogeneity***_
Overall	0.99 (0.97-1.01)	
Invasive	0.99 (0.97-1.01)	*0.78*
*In situ*	0.98 (0.92-1.04)	
Invasive ER+	0.98 (0.95-1.00)	*0.08*
Invasive ER-	1.03 (0.98-1.10)	
Invasive PR+	0.99 (0.96-1.02)	*0.91*
Invasive PR-	0.99 (0.95-1.04)	
Invasive HER2+	1.04 (0.96-1.12)	*0.15*
Invasive HER2-	0.97 (0.93-1.01)	

*CI* Confidence Interval, *ER* Estrogen Receptor, *HER2* Human epidermal growth factor receptor 2, *HR* Hazard ratio, *NOVA 3* Processed foods, *PR* Progesterone receptor, *SD* Standard deviation

^a^Models were stratified by age and center and adjusted for education, height, physical activity, age at menarche, oral contraceptive use, age at first full-term pregnancy, parity, breastfeeding, menopausal status, and menopausal hormone therapy use. Each NOVA group was mutually adjusted for the other NOVA groups

**Table 6:** Associations between NOVA intake (in g /d) and breast cancer risk, stratified by alcohol intake and BMI at recruitment

**Characteristics at recruitment**		**NOVA 1**	**NOVA 2**	**NOVA 3**	**NOVA 4**
	**N** **cases**	**HR per 1 SD (95%CI)**^a^	**HR per 1 SD (95%CI)**^a^	**HR per 1 SD (95%CI)**^a^	**HR per 1 SD (95%CI)**^a^
**Alcohol intake (g/d)**					
Non-drinkers	2014	0.99 (0.93-1.05)	1.00 (0.94-1.05)	1.00 (0.91-1.10)	1.01 (0.95-1.07)
> 0 - ≤3	4311	0.97(0.93-1.01)	1.03 (0.99-1.07)	1.00 (0.93-1.07)	1.01 (0.98-1.05)
>3 - ≤12	4594	1.02 (0.98-1.06)	1.02 (0.98-1.06)	0.99 (0.93-1.05)	1.00 (0.97-1.04)
>12 - ≤24	2298	0.98 (0.93-1.03)	0.97 (0.92-1.03)	1.05 (0.99-1.12)	1.01 (0.96-1.07)
>24	1716	0.99 (0.93-1.05)	0.99 (0.93-1.05)	1.02 (0.99-1.06)	1.01(0.95-1.07)
*P*_*interaction*_		*0.79*	*0.17*	*0.68*	*0.52*
**Body mass index**					
<25	8871	0.99 (0.97-1.02)	1.01 (0.98-1.04)	1.05 (1.03-1.08)	1.01 (0.98-1.04)
≥25	6062	0.97 (0.94-1.01)	1.01 (0.97-1.04)	1.05 (1.02-1.08)	1.00 (0.97-1.03)
*P*_*interaction*_		*0.57*	*0.31*	*0.27*	*0.88*

*CI* Confidence Interval, *NOVA 1* Unprocessed/minimally processed foods, *NOVA 2* Processed culinary ingredients, *NOVA 3* Processed foods, *NOVA 4* Ultra-processed foods, *SD* Standard deviation

^a^Models were stratified by age and center and adjusted for education, height, physical activity, age at menarche, oral contraceptive use, age at first full-term pregnancy, parity, breastfeeding, menopausal status and menopausal hormone therapy use. Each NOVA group was mutually adjusted for the other NOVA groups

In secondary analyses using %g/d, kcal/d or %kcal/d as the exposure, the results were consistent with those obtained in the main analyses (Supplementary Table S7). However, when using %g/d as an exposure variable, a higher intake of NOVA 1 was associated with a slightly lower risk of breast cancer [HR_per 1 SD_ =0.96 (0.94 - 0.98)]. Nevertheless, this association was no longer statistically significant when the model was further adjusted for alcohol intake [HR_per 1 SD_ =0.98 (0.94-1.00), Supplementary Table S7]. In addition, the results were similar when we used lower and upper bound scenarios (data not shown). Finally, no heterogeneity was reported by country (P_homogeneity_ ≥ 0.09, Supplementary Table S8).

## Discussion

In this large-scale prospective analysis, we found a positive association between the consumption of processed foods and breast cancer, which was likely driven by alcohol – an already established risk factor for breast cancer. The association between the degree of food processing and breast cancer risk did not differ by breast cancer subtype, menopausal status, alcohol intake or BMI.

In this study, no associations were found between the consumption of food included in the NOVA 1 group and breast cancer risk, overall or by breast cancer subtypes when the absolute values of intake were evaluated (g/day or kcal/day). Although a slight inverse association was reported when the %g/day values were used, this association disappeared when models were further adjusted for alcohol intake. Furthermore, because we only observed an inverse association when the variable was expressed as %g/d, these results might be because individuals who consumed more food from NOVA group 1 also consumed less food from NOVA group 3. Only one cohort study (Fiolet et al., 2018) and one case-control study (Jacobs et al., 2022) investigated associations between NOVA 1 and breast cancer risk and reported an inverse association (using %g/d and %kcal/d, respectively). Although NOVA 1 foods have low energy density and are rich in phytochemicals (carotenoids, flavonoids, dietary fiber), vitamins and minerals, known to be anticancerogenic (Bakker et al., 2016), our study does not support the hypothesis of a lower breast cancer risk with higher intake of NOVA 1 foods. In addition, we found no evidence of an association between NOVA 2 and breast cancer risk, as also reported in a South African case-control study (Jacobs et al., 2022).

We observed a positive association between NOVA 3 intake and breast cancer risk. To our knowledge, no previous population study has reported a positive association between NOVA 3 and breast cancer risk (Fiolet et al., 2018; Jacobs et al., 2022). Interestingly, in our study population, the positive association disappeared when models were adjusted for alcohol consumption or when alcoholic drinks were excluded from the NOVA groups. Indeed, alcohol is an established risk factor for breast cancer and, as such, could drive the positive association between processed foods and breast cancer risk. Of note, in this study we observed that, on average, beer and wine made up 35% of NOVA 3 g/day intake.

Furthermore, we found no evidence of an association between the consumption of NOVA 4 and breast cancer risk. Other studies have also reported no association between NOVA 4 intake and breast cancer risk (Jacobs et al., 2022; Romaguera et al., 2021). However, our results differ from those from the NutriNet-Santé French cohort and two case-control studies, which reported a positive association between the consumption of NOVA 4 and breast cancer risk (Fiolet et al., 2018; Queiroz et al., 2018; Romieu et al., 2022). It has been suggested that NOVA 4 foods may increase breast cancer risk through several factors such as their high energy density due to added sugars and fats, the presence of a variety of additives, preservatives and processing contaminants (e.g. acrylamide, *trans*-fatty acids, endocrine disrupters, etc.) or lack of fiber, proteins, and other components that are associated with fullness and satisfaction, leading individuals to eat more in an attempt to feel satisfied/saturated (Friedman, 2015; Luiten et al., 2016; Moubarac et al., 2013; Pouzou et al., 2018). In addition, we might have expected to observe a positive association between NOVA 4 and breast cancer risk due to alcoholic distilled drinks, however, in this study population alcoholic distilled drinks made up 2.2% of NOVA 4 g/day intake. The lack of association between NOVA 4 and breast cancer risk in the current study might be explained by the fact that the consumption of NOVA 4 in EPIC was quite low as this was based on dietary intakes at recruitment (during the nineties); since then, the consumption of NOVA 4 has replaced the consumption of other NOVA groups (e.g. recipes that were made at home in the 1990s may currently be industrially processed), which may bias the associations with breast cancer risk. Indeed, NOVA 4 has been recently suggested to represent up to 60% of total daily energy intake in some countries of the European area such as the UK (Rauber et al., 2018) while in the current EPIC study population, NOVA 4 contributed to 31% of total energy intake (data not shown). However, when we classified food products based on the modern/current food environment (upper bound scenario), the association between NOVA 4 and breast cancer risk was still null.

Finally, in our study, the associations between the degree of food processing and breast cancer risk did not differ by invasiveness and hormone receptor status. Our results are consistent with a previous study that investigated the association between NOVA 4 and breast cancer risk by molecular status suggesting no differences in risk estimates [ER+ or PR+: OR_10% increase_=1.04 (0.96 – 1.13); HER2+: OR_10% increase_=0.96 (0.84 – 1.10); ER-PR-HER2-: OR_10% increase_=0.93 (0.75 – 1.15)] (Romaguera et al., 2021). However, another study reported a positive association between NOVA 4 and ER+ breast cancer [OR_T3vsT1_=2.44 (1.01 - 5.90)] and no clear association with ER- breast cancer [OR_T3vsT1_=1.87 (0.43 - 8.13)] (Romieu et al., 2022). No other studies reported results for other NOVA groups by invasiveness and molecular status of breast cancer, which makes any comparison with our results challenging.

Our study has several strengths including the prospective design, the multicenter aspect, the long-term follow-up, and the availability of a comprehensive assessment of participant characteristics, as well as the large number of incident breast cancer cases. We had self-reported data on lifestyle, reproductive, and medical factors and were therefore able to consider a wide range of potential confounders. We also stratified the analyses according to menopausal status, alcohol intake, obesity breast cancer subtype. However, it should be noted that the statistical power for some of these stratified analyses was rather low (e.g. for some of the breast cancer subtypes), therefore, these results should be interpreted with caution. The major limitation of the study is that dietary data were only collected at baseline (in the 1990s) while the food environment changed in the intervening years, exposing the EPIC participants to potentially different degrees of food processing over the course of their follow-up. However, three different scenarios were created considering that the food environment may have changed over time compared to the baseline. The lower and upper bound scenarios were used in sensitivity analyses to explore the potential impact of further industrialization of food products and of changes in consumer habits to convenience foods over time, and results were virtually unchanged. The fact that dietary data were collected only once may cause random measurement error and may fail to reflect long-term habits; any such bias would likely lead to an underestimation of true associations (i.e. regression dilution bias) (Clarke et al., 1999; Hutcheon et al., 2010). However, it is noteworthy that recent analyses comparing dietary follow-up data in some of the EPIC countries demonstrate only minor changes in dietary intakes among the EPIC participants, potentially due to the relatively older age of the participants included in the cohort (unpublished data). Finally, the dietary questionnaires used in EPIC were not designed to identify different food processing categories. Therefore, several assumptions had to be made when insufficient information about the processing of the food item was available, potentially contributing to measurement error. However, byproducts of processing (e.g. *trans* fatty acids or syringol metabolites) have been positively associated with NOVA group 4 in EPIC which is a sign of a good measurement of ultra-processed foods in EPIC (Huybrechts et al., 2022).

## Conclusion

This large-scale prospective analysis among European women suggests that the positive association between processed food intake and breast cancer risk was likely driven by alcoholic beverage consumption. Other degrees of food processing were not associated with breast cancer risk.

## Supplementary Information

Supplementary Material 1.

## Data Availability

For information on how to apply for gaining access to EPIC data and/or biospecimens, please follow the instructions at https://epic.iarc.fr/access/submit_appl_access.php.

## Abbreviations

BMI: Body Mass Index
CIs: Confidence Intervals
EPIC: European Prospective Investigation into Cancer and Nutrition
ER: Estrogen Receptor
HER2: Human Epidermal Growth Factor Receptor 2
HRs: Hazard Ratios
IARC: International Agency for Research on Cancer
ICD-O: International Classification of Diseases for Oncology
MHT: Menopausal Hormone Therapy
OR: Odd Ratio
PR: Progesterone Receptor
SD: Standard Deviation
