# Supplementary material for: Degree of food processing and breast cancer risk: a prospective study in 9 European countries
Source: Food Prod Process Nutr. 2024 Oct 9;6(1):89. doi: 10.1186/s43014-024-00264-2 (PMC11468235; doi:10.1186/s43014-024-00264-2)
Supplement: Supplementary file 1 — Supplementary Material 1. [file 43014_2024_264_MOESM1_ESM.docx]

**ADDITIONAL FILE 1. Supplementary Text, Tables and Figures**

**Degree of food processing and breast cancer risk: a prospective study in 9 European countries**

| **Text S1**. Definition of NOVA classification | Page 2 |
| --- | --- |
| **Table S1**. Associations between 1-SD increment of NOVA groups (in g/d) and breast cancer risk | Page 3 |
| **Table S2**. Baseline characteristics of participants, overall and according to quartiles of NOVA 1 intake (g/day). | Page 4 |
| **Table S3**. Baseline characteristics of participants, overall and according to quartiles of NOVA 2 intake (g/day). | Page 6 |
| **Table S4**. Baseline characteristics of participants, overall and according to quartiles of NOVA 3 intake (g/day). | Page 8 |
| **Table S5.** Baseline characteristics of participants, overall and according to quartiles of NOVA 4 intake (g/day). | Page 10 |
| **Table S6**. Associations between 1-SD increment of NOVA groups (in g/d) and breast cancer risk, overall and by breast cancer subtypes and stratified by menopausal status | Page 12 |
| **Table S7**. Associations between NOVA intake (% g/d, Kcal/d or %kcal/d) and overall breast cancer risk. | Page 13 |
| **Table S8**. Associations between NOVA groups intake (in g/d) and breast cancer risk by country. | Page 14 |
| **Figure S1:** Flow-chart of the study population | Page 16 |

**Text S1. Definition of NOVA classification**

Unprocessed or minimally processed foods (NOVA group 1) are natural foods (e.g. fresh plants/fruits/vegetables or parts of animals after separation from nature), or natural foods altered by methods such as freezing, pasteurization, fermentation, grinding, and other methods that do not include the addition of salt, sugar, oils or fats (e.g. dry or frozen plants/fruits/vegetables, grains, flour and pasta, fresh or frozen meat, milk, coffee and beans). Processed culinary ingredients (NOVA group 2) are substances derived from group 1 foods or directly from nature by processes including pressing, refining, grinding, milling and drying (e.g. oils, butter, sugar and salt). Processed foods (NOVA group 3) are products made by using preservation or cooking methods including canning and bottling (e.g. homemade breads, cheeses, beer, wine, and smoked fish). Ultra-processed foods (NOVA group 4) are industrial formulations manufactured from substances derived from foods or synthesized from other organic sources, using a series of industrial processes (e.g. processed meats, carbonated soft drinks, industrial packaged breads and buns, sweet or savory packaged snacks, chocolate, and ready-to-eat-meals). These type sof food might contain many additives (such as flavors, sweeteners, emulsifiers) and are usually packed in synthetic materials. Ultra-processed foods are made to be hyper-palatable and attractive, with long shelf-life, and able to be consumed anywhere, any time (Monteiro et al. 2019).

**References**

Monteiro CA, Cannon G, Levy RB, Moubarac J-C, Louzada ML, Rauber F, et al. Ultra-processed foods: what they are and how to identify them. Public Health Nutr. avr 2019;22(5):936‑41.

**Table S1.** **Associations between 1-SD increment of NOVA groups (in g/d) and breast cancer risk**

|  | **NOVA 1** | **NOVA 2** | **NOVA 3** | **NOVA 4** |
| --- | --- | --- | --- | --- |
| **Models** | HR (95%CI) | HR (95%CI) | HR (95%CI) | HR (95%CI) |
| Model 1* | 0.99 (0.97-1.01) | 1.01 (0.98-1.03) | 1.05 (1.03-1.07) | 1.01 (0.99-1.03) |
| Model 1 further adjusted for BMI | 0.98 (0.96-1.00) | 1.01 (0.99-1.03) | 1.05 (1.03-1.07) | 1.00 (0.98-1.02) |
| Model 1 further adjusted for mediteranean diet | 0.99 (0.97-1.01) | 1.01 (0.98-1.03) | 1.05 (1.03-1.07) | 1.00 (0.98-1.03) |
| Model 1 further adjusted for sodium intake | 1.00 (0.97-1.02) | 1.01 (0.99-1.03) | 1.06 (1.04-1.08) | 1.02 (0.99-1.04) |
| Model 1 further adjusted for carbohydrate intake | 1.00 (0.98-1.02) | 1.01 (0.99-1.04) | 1.06 (1.04-1.07) | 1.03 (1.00-1.05) |
| Model 1 further adjusted for lipid intake | 0.99 (0.97-1.01) | 1.01 (0.98-1.03) | 1.05 (1.03-1.07) | 1.01 (0.99-1.03) |
| Model 1 further adjusted for energy intake | 0.99 (0.97-1.02) | 1.01 (0.99-1.03) | 1.06 (1.03-1.08) | 1.02 (0.99-1.04) |
| Model 1 further adjusted for alcohol intake | 0.99 (0.97-1.01) | 1.01 (0.99-1.03) | 1.01 (0.98-1.03) | 1.01 (0.99-1.03) |

Note. CI, Confidence Interval. HR, Hazard ratio; NOVA 1, unprocessed/minimally processed foods; NOVA 2, processed culinary ingredients; NOVA 3, processed foods; NOVA 4, ultra-processed foods; SD, Standard deviation.

* Model 1 was stratified by age and centre and adjusted for education, height, physical activity, age at menarche, oral contraceptive use, age at first full-term pregnancy and parity, breastfeeding, menopausal status and menopausal hormone therapy use. Each NOVA group were mutually adjusted for the other NOVA groups.

**Table S2. Baseline characteristics of participants, overall and according to quartiles of NOVA 1 intake (g/day).**

|  | **Quarters of relative intake of minimally-processed food in diet (g/day)** | | | | | | | |
| --- | --- | --- | --- | --- | --- | --- | --- | --- |
|  | **Quartile 1, n= 79672** | | **Quartile 2, n= 79671** | | **Quartile 3, n= 79671** | | **Quartile 4, n= 79672** | |
|  | **N or Mean** | **% or SD** | **N or Mean** | **% or SD** | **N or Mean** | **% or SD** | **N or Mean** | **% or SD** |
| **Characteristics at recruitment** |  |  | | | |  |  |  |
| **Age (years)** | 48.39 | 8.9 | 49.38 | 10.13 | 51.76 | 10.22 | 53.3 | 8.5 |
| **Education status** |  |  |  |  |  |  |  |  |
| None | 6332 | 56.1 | 3886 | 34.43 | 820 | 7.27 | 248 | 2.2 |
| Primary school | 25134 | 34.26 | 18669 | 25.45 | 14241 | 19.41 | 15320 | 20.88 |
| Technical/professional school | 16797 | 23.62 | 17967 | 25.26 | 17885 | 25.15 | 18475 | 25.98 |
| Higher education | 30175 | 20.13 | 36078 | 24.07 | 41799 | 27.89 | 41817 | 27.9 |
| Unknown | 1234 | 9.46 | 3071 | 23.55 | 4926 | 37.77 | 3812 | 29.23 |
| **Height (cm)** | 161.59 | 7.17 | 162.48 | 6.61 | 162.89 | 6.26 | 163.27 | 6.12 |
| **BMI (kg/m^2^)** | 25.26 | 4.48 | 24.77 | 4.34 | 24.42 | 4.14 | 24.74 | 4.25 |
| **Age at menarche (years)** |  |  |  |  |  |  |  |  |
| ≤ 13 | 52450 | 25.83 | 50925 | 25.08 | 50144 | 24.69 | 49548 | 24.4 |
| > 13 | 27222 | 23.54 | 28746 | 24.86 | 29527 | 25.54 | 30124 | 26.05 |
| Unknown | 2697 | 25.13 | 3663 | 34.13 | 2684 | 25.01 | 1688 | 15.73 |
| **Oral contraceptives use** |  |  |  |  |  |  |  |  |
| Ever | 34357 | 28.44 | 29174 | 24.15 | 27975 | 23.16 | 29297 | 24.25 |
| Never | 43020 | 22.71 | 47283 | 24.96 | 49694 | 26.23 | 49458 | 26.11 |
| Unknown | 2295 | 27.23 | 3214 | 38.13 | 2002 | 23.75 | 917 | 10.88 |
| **Number of full term pregnancy** |  |  |  |  |  |  |  |  |
| Nulliparous | 10865 | 23.78 | 12252 | 26.82 | 12247 | 26.81 | 10317 | 22.58 |
| 1 or 2 children | 12611 | 26.82 | 11353 | 24.15 | 11246 | 23.92 | 11809 | 25.12 |
| >3 children | 21110 | 25.83 | 19923 | 24.38 | 19896 | 24.34 | 20799 | 25.45 |
| Unknown | 3789 | 16.74 | 6899 | 30.49 | 6864 | 30.33 | 5077 | 22.44 |
| **Age at first full term pregnancy (years), n missing=911** | 24.93 | 4.36 | 24.91 | 4.36 | 25.1 | 4.33 | 24.63 | 4.24 |
| **Breastfed** |  |  |  |  |  |  |  |  |
| Ever | 18797 | 23.46 | 19662 | 24.54 | 21261 | 26.53 | 20406 | 25.47 |
| Never | 54656 | 26.87 | 48525 | 23.85 | 48213 | 23.7 | 52038 | 25.58 |
| Unknown | 6219 | 17.7 | 11484 | 32.69 | 10197 | 29.03 | 7228 | 20.58 |
| **Menopausal status** |  |  |  |  |  |  |  |  |
| Premenopausal | 34578 | 31.24 | 32031 | 28.94 | 25309 | 22.87 | 18760 | 16.95 |
| Perimenopausal | 15696 | 25 | 15029 | 23.93 | 15171 | 24.16 | 16900 | 26.91 |
| Postmenopausal | 29398 | 20.24 | 32611 | 22.46 | 39191 | 26.99 | 44012 | 30.31 |
| **Age at menopause (years), n missing=** | 48.43 | 4.68 | 48.7 | 4.81 | 48.78 | 5.05 | 48.84 | 5.15 |
| **Use of MHT** |  |  |  |  |  |  |  |  |
| Ever | 60013 | 27.68 | 55412 | 25.56 | 52422 | 24.18 | 48947 | 22.58 |
| Never | 15753 | 19.62 | 17446 | 21.73 | 21238 | 26.45 | 25845 | 32.19 |
| Unknown | 3906 | 18.07 | 6813 | 31.53 | 6011 | 27.82 | 4880 | 22.58 |
| **Physical activity** |  |  |  |  |  |  |  |  |
| Inactive | 20777 | 31.99 | 17338 | 26.69 | 14656 | 22.56 | 12186 | 18.76 |
| Moderately inactive | 26481 | 24.23 | 27408 | 25.08 | 28226 | 25.83 | 27180 | 24.87 |
| Moderately active | 22744 | 25.69 | 22255 | 25.14 | 21556 | 24.35 | 21965 | 24.81 |
| Active | 7987 | 15.92 | 11057 | 22.04 | 13696 | 27.3 | 17423 | 34.73 |
| Unknown | 1683 | 29.26 | 1613 | 28.05 | 1537 | 26.73 | 918 | 15.96 |
| **Smoking status** |  |  |  |  |  |  |  |  |
| Never | 42970 | 24.54 | 44541 | 25.44 | 45191 | 25.81 | 42382 | 24.21 |
| Former | 16764 | 22.53 | 17540 | 23.58 | 19471 | 26.17 | 20625 | 27.72 |
| Current | 18387 | 29.58 | 16034 | 25.79 | 13109 | 21.09 | 14632 | 23.54 |
| Unknown | 1551 | 22.03 | 1556 | 22.1 | 1900 | 26.99 | 2033 | 28.88 |
| **Alcohol intake (g/day)** | 6.2 | 10.51 | 7.42 | 11.28 | 8.92 | 11.86 | 9.98 | 12.82 |
| **Mediterranean diet** |  |  |  |  |  |  |  |  |
| Low | 14222 | 21.06 | 18676 | 27.66 | 18193 | 26.95 | 16427 | 24.33 |
| Medium | 39222 | 25.11 | 37602 | 24.08 | 39305 | 25.17 | 40055 | 25.65 |
| High | 26228 | 27.61 | 23393 | 24.63 | 22173 | 23.34 | 23190 | 24.41 |
| **Total dietary energy intake (kcal/day)** | 1747.03 | 487.91 | 1944.46 | 508.8 | 2057.35 | 499.54 | 2236.77 | 566.28 |
| **Total fat (g/day)** | 67.5 | 23.63 | 75.05 | 26.03 | 78.88 | 26.07 | 84.63 | 28.99 |
| **Sodium (g/day)** | 1958.64 | 792.57 | 2230.56 | 917.88 | 2472.08 | 885.54 | 2949.75 | 989.87 |
| **Carbohydrate (g/day)** | 208.05 | 62.59 | 234.86 | 67.04 | 248.91 | 69.81 | 269.1 | 79.99 |
| **Intake of other NOVA groups (in g/d)** |  |  |  |  |  |  |  |  |
| Intake of NOVA 2 | 26.09 | 19.59 | 26.81 | 21.52 | 26.25 | 21.56 | 28.82 | 23.46 |
| Intake of NOVA 3 | 260.09 | 194.48 | 268.56 | 188.72 | 279.3 | 183.29 | 304.11 | 192.35 |
| Intake of NOVA 4 | 288.49 | 231.11 | 330.43 | 236.97 | 358.5 | 240.89 | 355.53 | 247.33 |

BMI, Body Mass Index; MHT, Menopausal Hormone Therapy; NOVA 1, unprocessed/minimally processed foods; NOVA 2, processed culinary ingredients; NOVA 3, processed foods; NOVA 4, ultra-processed foods; SD, Standard Deviation.

**Table S3. Baseline characteristics of participants, overall and according to quartiles of NOVA 2 intake (g/day).**

|  | **Quarters of relative intake of culinary ingredients in diet (g/day)** | | | | | | | |
| --- | --- | --- | --- | --- | --- | --- | --- | --- |
|  | **Quartile 1, n= 79,671** | | **Quartile 2, n= 79,672** | | **Quartile 3, n= 79,672** | | **Quartile 4, n= 79,672** | |
|  | **N or Mean** | **% or SD** | **N or Mean** | **% or SD** | **N or Mean** | **% or SD** | **N or Mean** | **% or SD** |
| **Characteristics at recruitment** |  |  | | | |  |  |  |
| **Age (years)** | 49.61 | 11.19 | 50.85 | 9.7 | 51.46 | 9.1 | 50.9 | 8.33 |
| **Education status** |  |  |  |  |  |  |  |  |
| None | 504 | 4.47 | 2308 | 20.45 | 4647 | 41.17 | 3827 | 33.91 |
| Primary school | 16897 | 23.03 | 16898 | 23.03 | 18624 | 25.39 | 20945 | 28.55 |
| Technical/professional school | 27077 | 38.07 | 22886 | 32.18 | 13064 | 18.37 | 8097 | 11.38 |
| Higher education | 29958 | 19.99 | 34771 | 23.2 | 40681 | 27.14 | 44459 | 29.67 |
| Unknown | 5235 | 40.14 | 2809 | 21.54 | 2656 | 20.36 | 2343 | 17.96 |
| **Height (cm)** | 163.97 | 6.22 | 163.66 | 6.54 | 161.55 | 6.66 | 161.04 | 6.41 |
| **BMI (kg/m^2^)** | 25.09 | 4.39 | 25.02 | 4.28 | 24.73 | 4.34 | 24.35 | 4.22 |
| **Age at menarche (years)** |  |  |  |  |  |  |  |  |
| ≤ 13 | 49611 | 24.43 | 49309 | 24.28 | 51534 | 25.38 | 52613 | 25.91 |
| > 13 | 30060 | 26 | 30363 | 26.26 | 28138 | 24.34 | 27058 | 23.4 |
| Unknown | 3657 | 34.08 | 3867 | 36.03 | 2133 | 19.88 | 1075 | 10.02 |
| **Oral contraceptives use** |  |  |  |  |  |  |  |  |
| Ever | 25755 | 21.32 | 27644 | 22.88 | 32839 | 27.18 | 34565 | 28.61 |
| Never | 51245 | 27.05 | 48914 | 25.82 | 45030 | 23.77 | 44266 | 23.36 |
| Unknown | 2671 | 31.69 | 3114 | 36.95 | 1803 | 21.39 | 840 | 9.97 |
| **Number of full-term pregnancy** |  |  |  |  |  |  |  |  |
| Nulliparous | 15169 | 33.21 | 11349 | 24.84 | 10392 | 22.75 | 8771 | 19.2 |
| 1 or 2 children | 10393 | 22.1 | 11431 | 24.31 | 12796 | 27.21 | 12399 | 26.37 |
| >3 children | 19377 | 23.71 | 20246 | 24.77 | 20472 | 25.05 | 21633 | 26.47 |
| Unknown | 6168 | 27.26 | 6583 | 29.09 | 5261 | 23.25 | 4617 | 20.4 |
| **Age at first full term pregnancy (years), n missing=911** | 24.57 | 4.43 | 24.78 | 4.39 | 25.06 | 4.28 | 25.11 | 4.18 |
| **Breastfed** |  |  |  |  |  |  |  |  |
| Ever | 21158 | 26.41 | 17330 | 21.63 | 20866 | 26.04 | 20772 | 25.92 |
| Never | 48214 | 23.7 | 51477 | 25.3 | 51017 | 25.08 | 52724 | 25.92 |
| Unknown | 10299 | 29.32 | 10865 | 30.93 | 7789 | 22.17 | 6175 | 17.58 |
| **Menopausal status** |  |  |  |  |  |  |  |  |
| Premenopausal | 29411 | 26.57 | 25960 | 23.46 | 26419 | 23.87 | 28888 | 26.1 |
| Perimenopausal | 14531 | 23.14 | 16340 | 26.02 | 15436 | 24.58 | 16489 | 26.26 |
| Postmenopausal | 35729 | 24.6 | 37372 | 25.74 | 37817 | 26.04 | 34294 | 23.62 |
| **Age at menopause (years), n missing=** | 48.33 | 5.22 | 48.72 | 4.91 | 48.92 | 4.83 | 48.86 | 4.83 |
| **Use of MHT** |  |  |  |  |  |  |  |  |
| Ever | 54228 | 25.01 | 50821 | 23.44 | 54000 | 24.91 | 57745 | 26.64 |
| Never | 20187 | 25.15 | 21571 | 26.87 | 20174 | 25.13 | 18350 | 22.86 |
| Unknown | 5256 | 24.32 | 7280 | 33.69 | 5498 | 25.44 | 3576 | 16.55 |
| **Physical activity** |  |  |  |  |  |  |  |  |
| Inactive | 12294 | 18.93 | 13106 | 20.18 | 18941 | 29.16 | 20616 | 31.74 |
| Moderately inactive | 25275 | 23.13 | 25358 | 23.2 | 28955 | 26.49 | 29707 | 27.18 |
| Moderately active | 24100 | 27.23 | 24378 | 27.54 | 20278 | 22.91 | 19764 | 22.33 |
| Active | 15607 | 31.11 | 14882 | 29.67 | 10649 | 21.23 | 9025 | 17.99 |
| Unknown | 2395 | 41.64 | 1948 | 33.87 | 849 | 14.76 | 559 | 9.72 |
| **Smoking status** |  |  |  |  |  |  |  |  |
| Never | 39131 | 22.35 | 41373 | 23.63 | 47201 | 26.96 | 47379 | 27.06 |
| Former | 21655 | 29.11 | 20621 | 27.72 | 16927 | 22.75 | 15197 | 20.43 |
| Current | 17389 | 27.97 | 16031 | 25.79 | 13704 | 22.05 | 15038 | 24.19 |
| Unknown | 1496 | 21.25 | 1647 | 23.39 | 1840 | 26.14 | 2057 | 29.22 |
| **Alcohol intake (g/day)** | 7 | 10.66 | 7.61 | 11.05 | 8.51 | 12.09 | 9.41 | 12.88 |
| **Mediterranean diet** |  |  |  |  |  |  |  |  |
| Low | 23048 | 34.14 | 20376 | 30.18 | 14177 | 21 | 9917 | 14.69 |
| Medium | 38826 | 24.86 | 42129 | 26.97 | 39130 | 25.05 | 36099 | 23.11 |
| High | 17797 | 18.74 | 17167 | 18.07 | 26365 | 27.76 | 33655 | 35.43 |
| **Total dietary energy intake (kcal/day)** | 1759.89 | 476.13 | 1883.56 | 487.93 | 1999.87 | 495.42 | 2342.3 | 543.11 |
| **Total fat (g/day)** | 61.32 | 21.82 | 70.04 | 22.47 | 78.45 | 23.26 | 96.25 | 26.89 |
| **Sodium (g/day)** | 2084.81 | 825.7 | 2326.36 | 971.92 | 2454.29 | 964.11 | 2745.57 | 991.25 |
| **Carbohydrate (g/day)** | 227.87 | 73.9 | 231.26 | 71.23 | 234.11 | 69.7 | 267.67 | 72.39 |
| **Intake of other NOVA groups (in g/d)** |  |  |  |  |  |  |  |  |
| Intake of NOVA 1 | 1958.42 | 818.22 | 1955.06 | 843.32 | 1953.43 | 854.67 | 2052.53 | 892.15 |
| Intake of NOVA 3 | 5.45 | 2.38 | 14.84 | 3.49 | 30.36 | 5.19 | 57.33 | 16.7 |
| Intake of NOVA 4 | 422.51 | 267.35 | 372.3 | 236.03 | 285.79 | 220.23 | 252.36 | 194.78 |

BMI, Body Mass Index; MHT, Menopausal Hormone Therapy; NOVA 1, unprocessed/minimally processed foods; NOVA 2, processed culinary ingredients; NOVA 3, processed foods; NOVA 4, ultra-processed foods; SD, Standard Deviation.

**Table S4. Baseline characteristics of participants, overall and according to quartiles of NOVA 3 intake (g/day).**

|  | **Quarters of relative intake of processed food in diet (g/day)** | | | | | | | |
| --- | --- | --- | --- | --- | --- | --- | --- | --- |
|  | **Quartile 1, n= 79,671** | | **Quartile 2, n= 79,672** | | **Quartile 3, n= 79,672** | | **Quartile 4, n= 79,672** | |
|  | **N or Mean** | **% or SD** | **N or Mean** | **% or SD** | **N or Mean** | **% or SD** | **N or Mean** | **% or SD** |
| **Characteristics at recruitment** |  |  | | | |  |  |  |
| **Age (years)** | 50.05 | 10.5 | 50.83 | 9.92 | 50.92 | 9.47 | 51.03 | 8.63 |
| **Education status** |  |  |  |  |  |  |  |  |
| None | 2915 | 25.83 | 3251 | 28.81 | 3018 | 26.74 | 2102 | 18.62 |
| Primary school | 19109 | 26.05 | 18095 | 24.66 | 18220 | 24.84 | 17940 | 24.45 |
| Technical/professional school | 21891 | 30.78 | 19322 | 27.17 | 16355 | 23 | 13556 | 19.06 |
| Higher education | 29680 | 19.8 | 35766 | 23.86 | 40029 | 26.71 | 44394 | 29.62 |
| Unknown | 6076 | 46.58 | 3238 | 24.83 | 2050 | 15.72 | 1679 | 12.87 |
| **BMI (kg/m^2^)** | 25.15 | 4.53 | 24.94 | 4.34 | 24.69 | 4.26 | 24.41 | 4.09 |
| **Age at menarche (years)** |  |  |  |  |  |  |  |  |
| ≤ 13 | 50857 | 25.04 | 50333 | 24.79 | 50504 | 24.87 | 51373 | 25.3 |
| > 13 | 28814 | 24.92 | 29339 | 25.38 | 29168 | 25.23 | 28298 | 24.48 |
| Unknown | 4557 | 42.46 | 3478 | 32.41 | 1803 | 16.8 | 894 | 8.33 |
| **Oral contraceptives use** |  |  |  |  |  |  |  |  |
| Ever | 30967 | 25.63 | 29933 | 24.78 | 29954 | 24.8 | 29949 | 24.79 |
| Never | 44976 | 23.74 | 46928 | 24.77 | 48350 | 25.52 | 49201 | 25.97 |
| Unknown | 3728 | 44.23 | 2811 | 33.35 | 1368 | 16.23 | 521 | 6.18 |
| **Number of full-term pregnancies** |  |  |  |  |  |  |  |  |
| Nulliparous | 12231 | 26.77 | 11057 | 24.2 | 11139 | 24.38 | 11254 | 24.64 |
| 1 or 2 children | 10500 | 22.33 | 10687 | 22.73 | 12279 | 26.11 | 13553 | 28.82 |
| >3 children | 21954 | 26.86 | 21295 | 26.06 | 19659 | 24.05 | 18820 | 23.03 |
| Unknown | 5975 | 26.4 | 6855 | 30.29 | 5746 | 25.39 | 4053 | 17.91 |
| **Age at first full term pregnancy (years), n missing=911** | 24.52 | 4.43 | 24.95 | 4.31 | 24.98 | 4.25 | 25.1 | 4.28 |
| **Breastfed** |  |  |  |  |  |  |  |  |
| Ever | 19796 | 24.71 | 18425 | 23 | 20157 | 25.16 | 21748 | 27.14 |
| Never | 49480 | 24.32 | 50483 | 24.82 | 51142 | 25.14 | 52327 | 25.72 |
| Unknown | 10395 | 29.59 | 10764 | 30.64 | 8373 | 23.84 | 5596 | 15.93 |
| **Menopausal status** |  |  |  |  |  |  |  |  |
| Premenopausal | 28434 | 25.69 | 27263 | 24.63 | 27520 | 24.86 | 27461 | 24.81 |
| Perimenopausal | 15908 | 25.33 | 15401 | 24.53 | 15476 | 24.64 | 16011 | 25.5 |
| Postmenopausal | 35329 | 24.33 | 37008 | 25.49 | 36676 | 25.26 | 36199 | 24.93 |
| **Age at menopause (years), n missing=** | 48.2 | 5.22 | 48.64 | 5.02 | 48.95 | 4.77 | 48.99 | 4.78 |
| **Use of MHT** |  |  |  |  |  |  |  |  |
| Ever | 55742 | 25.71 | 54937 | 25.34 | 53345 | 24.61 | 52770 | 24.34 |
| Never | 19940 | 24.84 | 19470 | 24.25 | 19786 | 24.65 | 21086 | 26.26 |
| Unknown | 3989 | 18.46 | 5265 | 24.36 | 6541 | 30.27 | 5815 | 26.91 |
| **Physical activity** |  |  |  |  |  |  |  |  |
| Inactive | 16398 | 25.24 | 15427 | 23.75 | 16088 | 24.77 | 17044 | 26.24 |
| Moderately inactive | 25026 | 22.9 | 26281 | 24.05 | 28387 | 25.97 | 29601 | 27.08 |
| Moderately active | 24535 | 27.72 | 22834 | 25.8 | 20777 | 23.47 | 20374 | 23.02 |
| Active | 11442 | 22.81 | 13436 | 26.78 | 13243 | 26.4 | 12042 | 24.01 |
| Unknown | 2270 | 39.47 | 1694 | 29.46 | 1177 | 20.47 | 610 | 10.61 |
| **Smoking status** |  |  |  |  |  |  |  |  |
| Never | 42303 | 24.16 | 44383 | 25.35 | 46039 | 26.3 | 42359 | 24.19 |
| Former | 18491 | 24.85 | 18940 | 25.46 | 18157 | 24.4 | 18812 | 25.28 |
| Current | 16757 | 26.96 | 14649 | 23.57 | 13956 | 22.45 | 16800 | 27.03 |
| Unknown | 2120 | 30.11 | 1700 | 24.15 | 1520 | 21.59 | 1700 | 24.15 |
| **Alcohol intake (g/day)** | 1.93 | 3.44 | 4.17 | 5.13 | 6.72 | 6.77 | 19.7 | 16.65 |
| **Mediterranean diet** |  |  |  |  |  |  |  |  |
| Low | 21401 | 31.7 | 19516 | 28.9 | 14145 | 20.95 | 12456 | 18.45 |
| Medium | 41788 | 26.76 | 38327 | 24.54 | 38245 | 24.49 | 37824 | 24.22 |
| High | 16482 | 17.35 | 21829 | 22.98 | 27282 | 28.72 | 29391 | 30.94 |
| **Total dietary energy intake (kcal/day)** | 1658.72 | 444.52 | 1883.34 | 449.09 | 2072.75 | 466.95 | 2370.8 | 550.81 |
| **Total fat (g/day)** | 60.62 | 21.08 | 72.41 | 22.51 | 82.07 | 24.59 | 90.96 | 29.09 |
| **Sodium (g/day)** | 1759.89 | 635 | 2192.15 | 729.37 | 2623.2 | 867.68 | 3035.79 | 1081.93 |
| **Carbohydrate (g/day)** | 215.6 | 71.15 | 232.44 | 68.66 | 246.42 | 67.9 | 266.47 | 76.66 |
| **Intake of other NOVA groups (in g/d)** |  |  |  |  |  |  |  |  |
| Intake of NOVA 1 | 1774.46 | 785.43 | 1997.05 | 829.72 | 2081.84 | 862.16 | 2066.1 | 897.29 |
| Intake of NOVA 2 | 16.57 | 15.9 | 23.1 | 19.06 | 30.82 | 21.36 | 37.49 | 23.38 |
| Intake of NOVA 4 | 371.2 | 260.15 | 345.94 | 240.35 | 320.38 | 233.77 | 295.42 | 220.5 |

BMI, Body Mass Index; MHT, Menopausal Hormone Therapy; NOVA 1, unprocessed/minimally processed foods; NOVA 2, processed culinary ingredients; NOVA 3, processed foods; NOVA 4, ultra-processed foods; SD, Standard Deviation.

**Table S5. Baseline characteristics of participants, overall and according to quartiles of NOVA 4 intake (g/day).**

|  | **Quarters of relative intake of ultra-processed food in diet (g/day)** | | | | | | | | | | |  |
| --- | --- | --- | --- | --- | --- | --- | --- | --- | --- | --- | --- | --- |
|  | **Quartile 1, n= 79672** | | **Quartile 2, n= 79671** | | **Quartile 3, n= 79671** | | | | **Quartile 4, n= 79672** | | | |
|  | **N or Mean** | **% or SD** | **N or Mean** | **% or SD** | **N or Mean** | | **% or SD** | | **N or Mean** | | **% or SD** | |
| **Characteristics at recruitment** |  |  | | | |  | |  | |  | |  |
| **Age (years)** | 51.99 | 8.09 | 51.5 | 8.76 | 50.74 | | 9.6 | | 48.59 | | 11.5 | |
| **Education status** |  |  |  |  |  | |  | |  | |  | |
| None | 7447 | 65.98 | 2188 | 19.39 | 1034 | | 9.16 | | 617 | | 5.47 | |
| Primary school | 23286 | 31.74 | 18022 | 24.57 | 16401 | | 22.36 | | 15655 | | 21.34 | |
| Technical/professional school | 7045 | 9.91 | 16194 | 22.77 | 22272 | | 31.31 | | 25613 | | 36.01 | |
| Higher education | 40078 | 26.74 | 40820 | 27.24 | 36526 | | 24.37 | | 32445 | | 21.65 | |
| Unknown | 1815 | 13.92 | 2448 | 18.77 | 3439 | | 26.37 | | 5341 | | 40.95 | |
| **Height (cm)** | 159.81 | 6.39 | 162.54 | 6.34 | 163.72 | | 6.34 | | 164.16 | | 6.37 | |
| **BMI (kg/m^2^)** | 25.03 | 4.46 | 24.51 | 4.1 | 24.61 | | 4.1 | | 25.04 | | 4.56 | |
| **Age at menarche (years)** |  |  |  |  |  | |  | |  | |  | |
| ≤ 13 | 53933 | 26.56 | 51089 | 25.16 | 48938 | | 24.1 | | 49107 | | 24.18 | |
| > 13 | 25738 | 22.26 | 28583 | 24.72 | 30734 | | 26.58 | | 30564 | | 26.44 | |
| Unknown | 2073 | 19.32 | 3466 | 32.3 | 2789 | | 25.99 | | 2404 | | 22.4 | |
| **Oral contraceptives use** |  |  |  |  |  | |  | |  | |  | |
| Ever | 38995 | 32.28 | 29844 | 24.7 | 27379 | | 22.66 | | 24585 | | 20.35 | |
| Never | 38767 | 20.46 | 46932 | 24.77 | 50339 | | 26.57 | | 53417 | | 28.2 | |
| Unknown | 1909 | 22.65 | 2896 | 34.36 | 1954 | | 23.18 | | 1669 | | 19.8 | |
| **Number of full term pregnancy** |  |  |  |  |  | |  | |  | |  | |
| Nulliparous | 9021 | 19.75 | 10051 | 22 | 11489 | | 25.15 | | 15120 | | 33.1 | |
| 1 or 2 children | 12372 | 26.31 | 11945 | 25.4 | 11295 | | 24.02 | | 11407 | | 24.26 | |
| >3 children | 22870 | 27.98 | 20225 | 24.75 | 20138 | | 24.64 | | 18495 | | 22.63 | |
| Unknown | 4539 | 20.06 | 6272 | 27.72 | 5790 | | 25.59 | | 6028 | | 26.64 | |
| **Age at first full term pregnancy (years), n missing=911** | 25.08 | 4.15 | 24.85 | 4.27 | 24.88 | | 4.39 | | 24.73 | | 4.49 | |
| **Breastfed** |  |  |  |  |  | |  | |  | |  | |
| Ever | 20732 | 25.87 | 19600 | 24.46 | 18604 | | 23.22 | | 21190 | | 26.45 | |
| Never | 51178 | 25.16 | 50340 | 24.75 | 52608 | | 25.86 | | 49306 | | 24.24 | |
| Unknown | 7761 | 22.09 | 9732 | 27.7 | 8460 | | 24.08 | | 9175 | | 26.12 | |
| **Menopausal status** |  |  |  |  |  | |  | |  | |  | |
| Premenopausal | 25019 | 22.61 | 25093 | 22.67 | 27203 | | 24.58 | | 33363 | | 30.14 | |
| Perimenopausal | 15491 | 24.67 | 17314 | 27.57 | 16399 | | 26.11 | | 13592 | | 21.64 | |
| Postmenopausal | 39161 | 26.97 | 37265 | 25.66 | 36070 | | 24.84 | | 32716 | | 22.53 | |
| **Age at menopause (years), n missing=** | 48.79 | 4.93 | 48.84 | 4.87 | 48.71 | | 4.95 | | 48.47 | | 5.08 | |
| **Use of MHT** |  |  |  |  |  | |  | |  | |  | |
| Ever | 57386 | 26.47 | 52405 | 24.17 | 52606 | | 24.27 | | 54397 | | 25.09 | |
| Never | 18522 | 23.07 | 21038 | 26.21 | 21222 | | 26.43 | | 19500 | | 24.29 | |
| Unknown | 3763 | 17.41 | 6229 | 28.82 | 5844 | | 27.04 | | 5774 | | 26.72 | |
| **Physical activity** |  |  |  |  |  | |  | |  | |  | |
| Inactive | 25021 | 38.52 | 14902 | 22.94 | 12126 | | 18.67 | | 12908 | | 19.87 | |
| Moderately inactive | 29540 | 27.03 | 28411 | 25.99 | 26043 | | 23.83 | | 25301 | | 23.15 | |
| Moderately active | 17431 | 19.69 | 23017 | 26 | 25009 | | 28.25 | | 23063 | | 26.05 | |
| Active | 7413 | 14.78 | 12111 | 24.14 | 14451 | | 28.81 | | 16188 | | 32.27 | |
| Unknown | 266 | 4.63 | 1231 | 21.4 | 2043 | | 35.52 | | 2211 | | 38.45 | |
| **Smoking status** |  |  |  |  |  | |  | |  | |  | |
| Never | 48958 | 27.96 | 43689 | 24.95 | 41148 | | 23.5 | | 41289 | | 23.58 | |
| Former | 14749 | 19.82 | 18962 | 25.49 | 20743 | | 27.88 | | 19946 | | 26.81 | |
| Current | 14147 | 22.76 | 15056 | 24.22 | 15986 | | 25.72 | | 16973 | | 27.3 | |
| Unknown | 1817 | 25.81 | 1965 | 27.91 | 1795 | | 25.5 | | 1463 | | 20.78 | |
| **Alcohol intake (g/day)** | 7.91 | 12.32 | 8.64 | 12.03 | 8.23 | | 11.46 | | 7.76 | | 11.08 | |
| **Mediterranean diet** |  |  |  |  |  | |  | |  | |  | |
| Low | 6756 | 10.01 | 15649 | 23.18 | 20498 | | 30.36 | | 24615 | | 36.46 | |
| Medium | 34166 | 21.88 | 41037 | 26.27 | 42154 | | 26.99 | | 38827 | | 24.86 | |
| High | 38749 | 40.8 | 22986 | 24.2 | 17020 | | 17.92 | | 16229 | | 17.09 | |
| **Total dietary energy intake (kcal/day)** | 1827.96 | 518.82 | 1891.23 | 531.08 | 1993.46 | | 484.87 | | 2272.96 | | 539.7 | |
| **Total fat (g/day)** | 71.36 | 24.66 | 73.82 | 27 | 76.54 | | 25.91 | | 84.35 | | 28.41 | |
| **Sodium (g/day)** | 2105.13 | 816.92 | 2313.12 | 891.73 | 2448.98 | | 931.83 | | 2743.81 | | 1104.39 | |
| **Carbohydrate (g/day)** | 211 | 66.54 | 220.28 | 63.43 | 239.39 | | 59.12 | | 290.25 | | 77.18 | |
| **Intake of other NOVA groups (in g/d)** |  |  |  |  |  | |  | |  | |  | |
| Intake of NOVA 1 | 1798.8 | 856.81 | 2015.35 | 854.93 | 2034.51 | | 843.3 | | 2070.79 | | 832.24 | |
| Intake of NOVA 2 | 36.83 | 20.63 | 28.38 | 22.14 | 22.54 | | 20.25 | | 20.22 | | 19.42 | |
| Intake of NOVA 3 | 310.31 | 196.21 | 290.42 | 196.42 | 260.68 | | 184.66 | | 250.64 | | 178.04 | |

BMI, Body Mass Index; MHT, Menopausal Hormone Therapy; NOVA 1, unprocessed/minimally processed foods; NOVA 2, processed culinary ingredients; NOVA 3, processed foods; NOVA 4, ultra-processed foods; SD, Standard Deviation.

**Table S6. Associations between 1-SD increment of NOVA groups (in g/d) and breast cancer risk, overall and by breast cancer subtypes and stratified by menopausal status.**

| **Breast cancer subtypes** | **N Cases** | **NOVA 1** | **NOVA 2** | **NOVA 3** | **NOVA 4** |
| --- | --- | --- | --- | --- | --- |
|  |  | HR (95%CI)^1^ | HR (95%CI)^1^ | HR (95%CI)^1^ | HR (95%CI)^1^ |
| **Premenopausal women** |  |  |  |  |  |
| Overall | 3797 | 0.95 (0.91-1.00) | 1.02 (0.98-1.06) | 1.04 (1.01-1.08) | 0.98 (0.94-1.02) |
| *In situ* | 481 | 0.89 (0.78-1.02) | 1.05 (0.94-1.18) | 1.01 (0.91-1.12) | 0.89 (0.79-1.01) |
| Invasive | 3314 | 0.96 (0.91-1.01) | 1.01 (0.97-1.06) | 1.05 (1.01-1.09) | 1.00 (0.96-1.04) |
| Invasive ER+ | 1987 | 0.95 (0.90-1.02) | 0.99 (0.94-1.05) | 1.06 (1.01-1.11) | 1.00 (0.95-1.06) |
| Invasive ER- | 478 | 1.01 (0.89-1.14) | 1.01 (0.91-1.13) | 1.05 (0.96-1.15) | 1.01 (0.90-1.13) |
| Invasive PR+ | 1520 | 0.99 (0.92-1.06) | 0.96 (0.90-1.02) | 1.07 (1.01-1.12) | 0.98 (0.91-1.05) |
| Invasive PR- | 654 | 0.91 (0.82-1.02) | 1.04 (0.95-1.14) | 1.05 (0.97-1.13) | 1.03 (0.93-1.14) |
| Invasive HER2+ | 288 | 1.00 (0.84-1.18) | 0.98 (0.85-1.13) | 0.98 (0.86-1.11) | 1.07 (0.94-1.21) |
| Invasive HER2- | 1096 | 0.93 (0.85-1.02) | 0.99 (0.92-1.06) | 1.07 (1.01-1.13) | 1.02 (0.95-1.09) |
| **Postmenopausal women** |  |  |  |  |  |
| Overall | 7731 | 1.00 (0.97-1.03) | 0.99 (0.97-1.02) | 1.06 (1.04-1.09) | 1.02 (0.99-1.04) |
| *In situ* | 722 | 1.00 (0.91-1.1) | 0.99 (0.9-1.09) | 1.08 (0.99-1.17) | 1.06 (0.97-1.15) |
| Invasive | 7002 | 1.00 (0.97-1.03) | 1.00 (0.97-1.03) | 1.06 (1.03-1.09) | 1.01 (0.98-1.04) |
| Invasive ER+ | 4118 | 1.00 (0.96-1.04) | 0.99 (0.95-1.03) | 1.07 (1.03-1.10) | 1.01 (0.97-1.05) |
| Invasive ER- | 851 | 1.03 (0.95-1.12) | 1.04 (0.95-1.13) | 1.11 (1.04-1.18) | 1.06 (0.98-1.14) |
| Invasive PR+ | 2556 | 0.99 (0.94-1.04) | 1.02 (0.97-1.07) | 1.05 (1.01-1.09) | 1.00 (0.95-1.06) |
| Invasive PR- | 1409 | 1.03 (0.96-1.10) | 0.95 (0.88-1.02) | 1.10 (1.05-1.16) | 1.04 (0.97-1.11) |
| Invasive HER2+ | 425 | 0.96 (0.85-1.09) | 1.01 (0.89-1.14) | 1.07 (0.98-1.18) | 0.98 (0.87-1.10) |
| Invasive HER2- | 1800 | 1.00 (0.94-1.06) | 1.01 (0.95-1.07) | 1.07 (1.02-1.12) | 1.04 (0.99-1.10) |

Note. CI, Confidence Interval; ER, Estrogen Receptor; HER2, human epidermal growth factor receptor 2; HR, Hazard ratio; NOVA 1, unprocessed/minimally processed foods; NOVA 2, processed culinary ingredients; NOVA 3, processed foods; NOVA 4, ultra-processed foods; PR, progesterone receptor; SD, Standard deviation.

^1^ Models were stratified by age and center and adjusted for education, height, physical activity, age at menarche, oral contraceptive use, age at first full-term pregnancy, parity, breastfeeding, menopausal status and menopausal hormone therapy use. Each NOVA group was mutually adjusted for the other NOVA groups.

**Table S7. Associations between NOVA intake (% g/d, Kcal/d or %kcal/d) and overall breast cancer risk.**

| **Units** | **Models** | **NOVA 1** | **NOVA 2** | **NOVA 3** | **NOVA 4** |
| --- | --- | --- | --- | --- | --- |
|  |  | HR (95%CI) | HR (95%CI) | HR (95%CI) | HR (95%CI) |
| %g/d | Model 1 | **0.96 (0.94-0.98)** | 1.02 (0.99-1.04) | **1.06 (1.04-1.08)** | 1.01 (0.98-1.03) |
|  | Model 2 | 0.98 (0.96-1.00) | 1.02 (1.00-1.05) | 1.02 (0.99-1.04) | 1.01 (0.99-1.03) |
|  |  |  |  |  |  |
|  | Model 1 | 1.00 (0.98-1.02) | 1.01 (0.99-1.04) | 1.02 (1.00-1.04) | 1.01 (0.99-1.03) |
| Kcal/d | Model 2 | 1.00 (0.98-1.02) | 1.01 (0.99-1.04) | 0.99 (0.97-1.01) | 1.01 (0.99-1.03) |
|  |  |  |  |  |  |
|  | Model 1 | 0.98 (0.96-1.00) | 1.00 (0.98-1.03) | 1.02 (1.00-1.04) | 1.00 (0.98-1.03) |
| %kcal/d | Model 2 | 0.99 (0.97-1.01) | 1.01 (0.98-1.03) | 0.99 (0.97-1.02) | 1.02 (0.99-1.04) |

Note. CI, Confidence Interval; HR, Hazard ratio; NOVA 1, unprocessed/minimally processed foods; NOVA 2, processed culinary ingredients; NOVA 3, processed foods; NOVA 4, ultra-processed foods; SD, Standard deviation.

Model 1 was stratified by age and centre and adjusted for education, height, physical activity, age at menarche, oral contraceptive use, age at first full-term pregnancy and parity, breastfeeding, menopausal status and menopausal hormone therapy use.

Model 2 was further adjusted for alcohol consumption. When using kcal/d, each NOVA group were mutually adjusted for the other NOVA groups.

**Table S8. Associations between NOVA groups intake (in g/d) and breast cancer risk by country**.

|  |  | **NOVA 1** | **NOVA 2** | **NOVA 3** | **NOVA 4** |
| --- | --- | --- | --- | --- | --- |
| **Countries** | **N cases** | **HR (95% CI)** | **95%CI** | **HR** | **95%CI** |
| **France** | 3821 | 0.99 (0.96-1.03) | 1.00 (0.97-1.04) | 1.05 (1.02-1.08) | 1.06 (1.00-1.12) |
| **Italy** | 1328 | 0.87 (0.75-1.02) | 1.03 (0.96-1.11) | 0.99 (0.94-1.05) | 1.01 (0.92-1.12) |
| **Spain** | 726 | 0.9 (0.75-1.08) | 1.13 (1.03-1.23) | 1.03 (0.95-1.13) | 1.00 (0.86-1.16) |
| **The United Kingdom** | 2157 | 1.00 (0.94-1.07) | 1.07 (1.01-1.13) | 1.06 (1.00-1.12) | 1.02 (0.98-1.06) |
| **The Netherlands** | 1191 | 0.96 (0.88-1.04) | 0.95 (0.88-1.03) | 1.14 (1.06-1.22) | 1.00 (0.92-1.08) |
| **Germany** | 915 | 1.00 (0.93-1.08) | 1.03 (0.96-1.10) | 1.04 (0.98-1.10) | 0.93 (0.87-1.00) |
| **Sweden** | 1433 | 0.95 (0.89-1.02) | 0.98 (0.92-1.05) | 1.06 (0.99-1.14) | 1.00 (0.92-1.08) |
| **Denmark** | 1992 | 1.02 (0.98-1.07) | 0.94 (0.87-1.02) | 1.05 (1.02-1.09) | 1.03 (0.99-1.07) |
| **Norway** | 1370 | 0.91 (0.8-1.04) | 0.93 (0.81-1.07) | 1.05 (0.91-1.21) | 0.91 (0.84-0.99) |
|  |  |  |  |  |  |
| ***P homogeneity*** |  | *0.95* | *0.14* | *0.63* | *0.09* |

Note. CI, Confidence Interval; HR, Hazard ratio; NOVA 1, unprocessed/minimally processed foods; NOVA 2, processed culinary ingredients; NOVA 3, processed foods; NOVA 4, ultra-processed foods; PR, progesterone receptor; SD, Standard deviation.

Models were stratified by age and centre and adjusted for education, height, physical activity, age at menarche, oral contraceptive use, age at first full-term pregnancy and parity, breastfeeding, menopausal status and menopausal hormone therapy use. Each NOVA group were mutually adjusted for the other NOVA groups.

**Figure S1:** Flow-chart of the study population

**Exclusion of participants from Greece**

26 048

**Exclusion of prevalent cancers**

25 184

**Exclusion of participants with no follow-up**

4 148

**Exclusion of participants with no lifestyle information or no dietary information**

6 259

**Exclusion of men**

131 425

**Exclusion of participants with extreme ranking on the ratio energy intake**

9 573

**EPIC participants**

521 323

**Final study population**

318 686
